# Clinical assessment of geometric distortion for a 0.35T MR‐guided radiotherapy system

**DOI:** 10.1002/acm2.13340

**Published:** 2021-07-07

**Authors:** John Neylon, Kiri A. Cook, Yingli Yang, Dongsu Du, Ke Sheng, Robert K. Chin, Amar U. Kishan, James M. Lamb, Daniel A. Low, Minsong Cao

**Affiliations:** ^1^ Department of Radiation Oncology University of California, Los Angeles (UCLA) Los Angeles CA USA; ^2^ Department of Radiation Medicine Oregon Health & Science University Oregon, Portland OR USA; ^3^ Department of Radiation Oncology City of Hope Cancer Center Los Angeles CA USA

**Keywords:** geometric distortion, MRgRT, MR‐guided radiotherapy, patient‐specific distortion

## Abstract

**Purpose:**

To estimate the overall spatial distortion on clinical patient images for a 0.35 T MR‐guided radiotherapy system.

**Methods:**

Ten patients with head‐and‐neck cancer underwent CT and MR simulations with identical immobilization. The MR images underwent the standard systematic distortion correction post‐processing. The images were rigidly registered and landmark‐based analysis was performed by an anatomical expert. Distortion was quantified using Euclidean distance between each landmark pair and tagged by tissue interface: bone‐tissue, soft tissue, or air‐tissue. For baseline comparisons, an anthropomorphic phantom was imaged and analyzed.

**Results:**

The average spatial discrepancy between CT and MR landmarks was 1.15 ± 1.14 mm for the phantom and 1.46 ± 1.78 mm for patients. The error histogram peaked at 0–1 mm. 66% of the discrepancies were <2 mm and 51% <1 mm. In the patient data, statistically significant differences (*p*‐values < 0.0001) were found between the different tissue interfaces with averages of 0.88 ± 1.24 mm, 2.01 ± 2.20 mm, and 1.41 ± 1.56 mm for the air/tissue, bone/tissue, and soft tissue, respectively. The distortion generally correlated with the in‐plane radial distance from the image center along the longitudinal axis of the MR.

**Conclusion:**

Spatial distortion remains in the MR images after systematic distortion corrections. Although the average errors were relatively small, large distortions observed at bone/tissue interfaces emphasize the need for quantitative methods for assessing and correcting patient‐specific spatial distortions.

## 1 **INTRODUCTION**:

Recent technological advances in MRI‐guided radiotherapy (MRgRT) systems have led to strong interest in incorporating MRI into the radiation therapy (RT) workflow due to its unique advantages.^1^, ^2^, ^3^ The superb soft‐tissue contrast of MR images enables the superior localization of the tumor and patient anatomy; therefore, it has the potential to improve patient setup accuracy and provide better guidance for adaptive radiotherapy. In addition, MR imaging does not expose patients to ionizing radiation, which is ideal for continuous real‐time imaging for tumor and organ motion tracking. However, one of the challenges of using MR in the RT workflow is its intrinsic geometric distortion caused by an imperfection in MR system hardware and change in the local magnetic field properties induced by the patient.^4^ Without correction, the geometric distortion can reach a few millimeters, which is larger than the spatial accuracy of 1mm recommended for stereotactic body radiation therapy by the American Association of Physicists in Medicine (AAPM) Task Group 142.^5^ This hinders MR application in the RT workflow, where spatial integrity needs to be maintained to a higher standard than diagnostic MRI.

In general, MR image geometric distortion can be classified as either system or patient‐dependent. System‐dependent distortion stems from the main static magnetic field (B0) inhomogeneity and gradient nonlinearity (GNL), while patient‐related distortion arises from differences in tissue magnetic susceptibilities and chemical shifts.^4^ For system‐related distortion, phantom‐based measurements can be used to characterize the distortion for a specific scanner and imaging sequence combination. Passive shimming and corrections applied during image reconstruction can be used to effectively minimize the system‐dependent distortion.^6^, ^7^ Systemic machine‐related distortions have been extensively assessed and reported for MR simulators and MRgRT systems with diverse system designs and various magnetic strengths.^8^, ^9^, ^10^, ^11^, ^12^, ^13^, ^14^, ^15^, ^16^, ^17^ GNL was found to be the primary source of system‐dependent distortion, resulting in a common pattern of increasing distortion with increasing distance from the image isocenter.^10^, ^15^ Moreover, patient‐related distortion is more difficult to assess and correct because it relies on the tissue magnetic properties of an individual subject. A simple way to estimate the patient‐related distortion is to simulate the susceptibility effects with a bulk susceptibility value assignment. However, this simulation‐based method requires prior knowledge of the susceptibility properties of the relevant tissues which can be difficult to obtain.^18^ B0 field mapping‐based methods can be used to directly measure the magnetic inhomogeneity induced by the imaged subject, but usually require additional image acquisition and post‐processing time.^19^, ^20^ To date, reports on the assessment of patient‐dependent geometric distortion for MR simulator and MRgRT system remain elusive. Stanescu et al. simulated the susceptibility distortions of several anatomical sites for magnetic field strengths from 0.5 to 3 T and large susceptibility distortions were identified at air‐tissue interfaces under magnets with higher field strength.^21^ Patient‐induced distortions were characterized for cranial images of a 3 T MR scanner by field mapping method and susceptibility‐induced distortions in the brain were found to be generally smaller than 2 mm.^19^ In a prospective study, landmark point‐based measurements were performed to evaluate the total distortions of head and neck (HN) cancer patients imaged by a 3 T MRI scanner in the same immobilization position as in the CTs^22^ and the overall distortions were quantified to be less than 2 mm. To the best of our knowledge, the comprehensive assessment of the spatial uncertainties including patient‐induced distortions of a low field strength MRgRT has not been reported.

The aim of this study was to assess the total geometric uncertainties including patient‐induced distortion of a 0.35 T MRgRT system by comparing MR images of an anthropomorphic phantom and clinical HN patients with corresponding CT scans acquired using the same immobilization position. Anatomic landmark point‐based measurements were performed between the corresponding MR and CT images to quantify the distortions with CT considered as the undistorted ground truth.

## 2 MATERIALS AND METHOD

As a baseline reference, an anthropomorphic triple modality 3D abdomen image fusion phantom (Model 057A, CIRS Inc. Norfolk, VA) was imaged to evaluate the distortion in a well‐controlled setting. The phantom simulates a small adult abdomen including various organs (lung, liver, kidneys, ribs, and vertebrae) with appropriate image contrast for CT, MRI, and ultrasound. Three MRI/CT multimodality markers (Beekley Medical Inc. Bristol, CT) were first placed on the phantom surface before it was scanned on a commercially available 0.35 T MR‐guided radiotherapy system (MRIdian, ViewRay Inc., Oakwood, OH) using the clinical balanced steady‐state free precession (bSSFP) sequence with vendor‐provided gradient offset (MRI‐GO) compensation to minimize system‐dependent distortion.^23^ The following MR acquisition parameters were used: field of view (FOV) of 50 × 45 × 43.2 cm^3^, 1.5 mm isotropic spatial resolution, and 172 sec acquisition time. The phantom was then scanned using a large‐bore 16‐slice CT scanner (Siemens SOMATOM Definition AS, Siemens Healthcare, Inc.) using the standard institutional simulation protocol (120 kVp/350 mAs, FOV 50 cm, 0.98 × 0.98 × 1.5 mm^3^ pixel size). The three MRI/CT multimodality markers were used in conjunction with laser systems to aid the phantom placement to ensure consistent positioning of the phantom between the MR and CT scans and minimize the uncertainty in image fusion.

Ten patients with newly diagnosed cancers of the head and neck were retrospectively selected from an IRB‐approved institutional registry trial investigating the feasibility and efficacy of MR‐guided radiotherapy. Details of patient immobilization and simulation were described in reference^24^ and summarized below. During the MRI simulation, patients were immobilized using a perforated, thermoplastic mask with the occiput supported on a modified Timo cushion (S‐type, Med‐Tec, Orange City, IA, USA), and scanned on the 0.35 T MR Linac system using the clinical bSSFP sequence. All the patients underwent CT simulation with the same immobilization devices on the same day of the MR simulation using the standard institutional CT simulation protocol described before. Both CT and MR image datasets which encompassed the patient from the apex of the skull to below the clavicles were then transferred into specialized radiotherapy software MIM (MIM Corporation, Cleveland OH) where the two scans were rigidly co‐registered.

**FIGURE 1:**
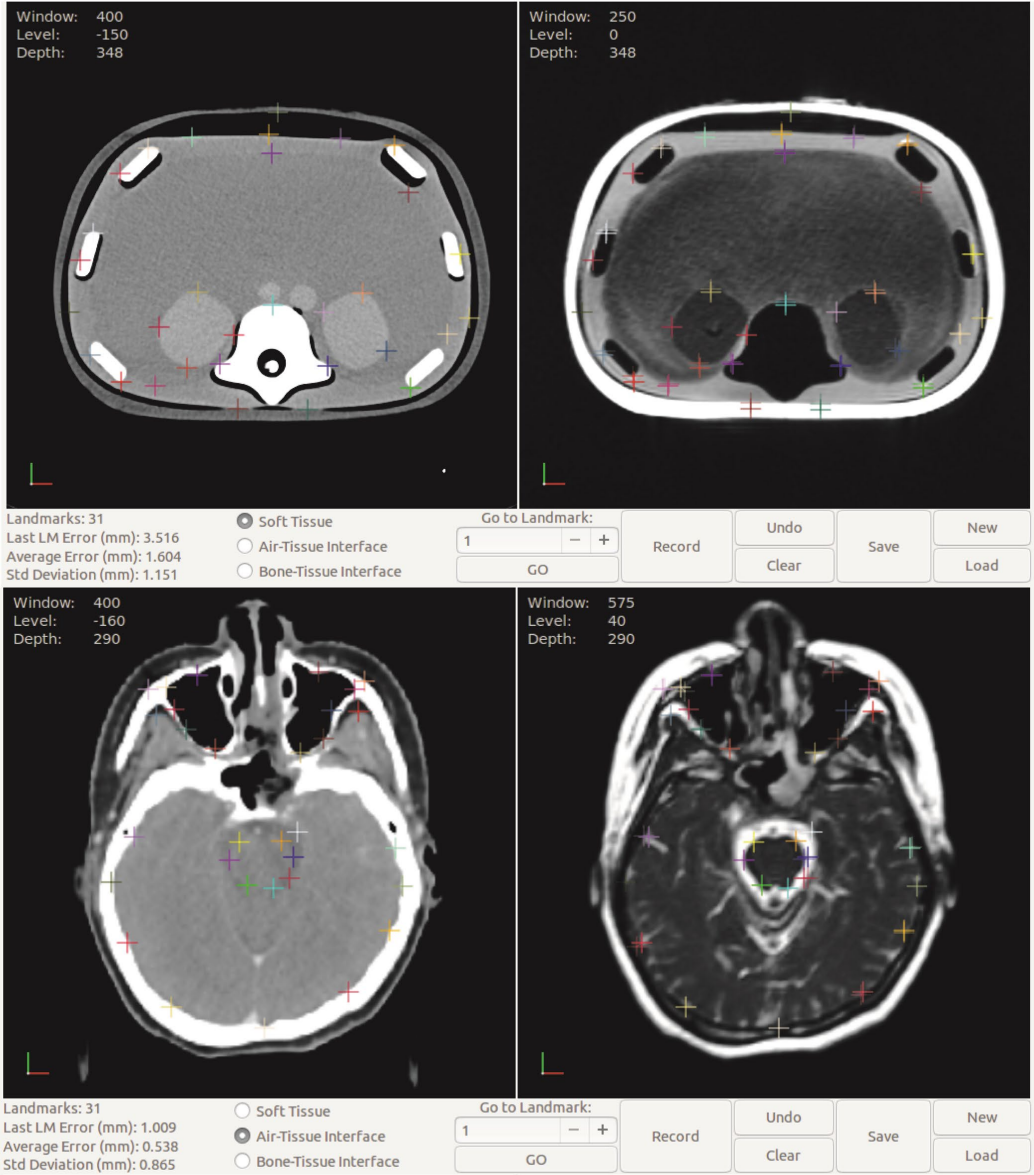
Landmark identification on an anthropomorphic phantom (top row) and patient images (bottom row) at various tissue interfaces using in‐house software. CT shown on left, MR with corrected landmarks and error vectors on right

In‐house software was developed for landmark‐based analysis, where the aligned CT and MR images were presented side‐by‐side, allowing an anatomical expert (radiation oncologist) to identify anatomical landmarks on both images. The software allowed the expert to zoom and pan the images, adjust the image window/level display according to clinical settings, and select an initial landmark on the CT image with sub‐voxel precision. A corresponding landmark would appear at the matching location on the MR image, at which point the expert could accept the corresponding landmark or apply a correction by selecting a different point on the MR image. The expert was also asked to classify each landmark pair by the type of an interface represented, either soft tissue to soft tissue, bone to soft tissue, or air/lung to soft tissue. Figure 1 shows the user interface of the in‐house software in which various landmarks were identified at different tissue interfaces on the anthropomorphic phantom. For the HN patient images, landmarks were identified in three representative axial levels through the middle of brainstem, center of maxillary sinus, and nasopharynx areas as well as C1‐C2 vertebral body. If necessary, rigid registration was adjusted locally at each level to minimize image registration uncertainty. For each anatomic landmark, the predicted and accepted positions were recorded and the Euclidean distance was calculated and used as the error between the points. Additional data recorded included two‐dimensional polar coordinates of each landmark with respect to the image center and the two‐dimensional polar coordinates of the error vector for each landmark with respect to its predicted position. Similar to the phantom images, landmarks were also tagged by the tissue interface.

## 3 RESULTS

**FIGURE 2:**
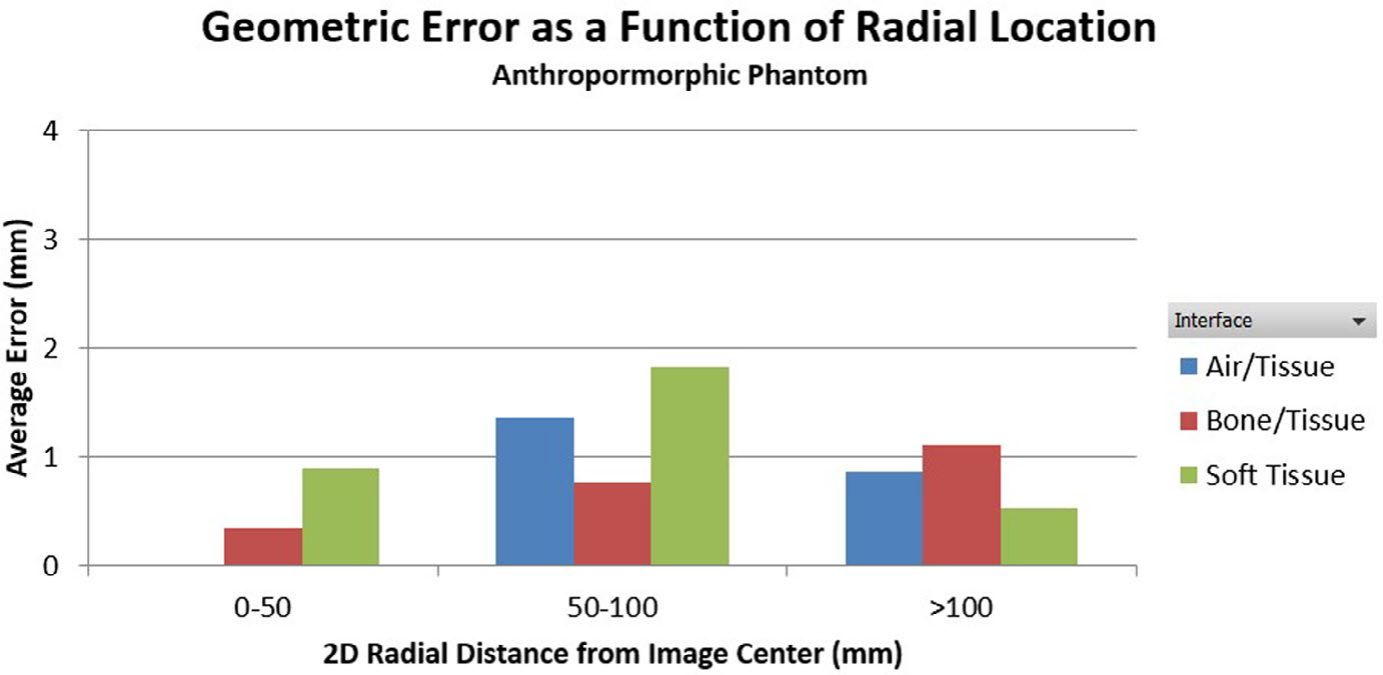
Average geometric error grouped by in‐plane radial distance from the image center for the anthropomorphic phantom. There are no air/tissue data points in the 0–50 mm region

A total of 92 landmarks were identified on the anthropomorphic phantom. As shown in Figure 1, these landmarks were localized at various tissue interface types including soft tissue interfaces such as boundaries of kidneys and liver, bone/tissue interfaces such as the surface of vertebrae or ribs, and air/tissue interfaces at lung and chest wall. The average spatial discrepancy between MR and CT was found to be 1.15 ± 1.14 mm for the anthropomorphic phantom. The average geometric errors were 1.21 ± 1.12 mm, 0.88 ± 1.02 mm, and 1.33 ± 1.24 mm at air/tissue, bone/tissue, and soft tissue interfaces, respectively. The average error was not significantly different between each type of interface (*p *= 0.24 for air/tissue vs. bone/tissue, *p *= 0.11 for tissue/tissue vs. bone/tissue, *p *= 0.70 soft tissue vs. air/tissue). The average geometric errors grouped by radial distance from the image center and tissue types are plotted in Figure 2. The maximum distortion was found to be 4.14mm, located at an air/tissue interface.

**FIGURE 3:**
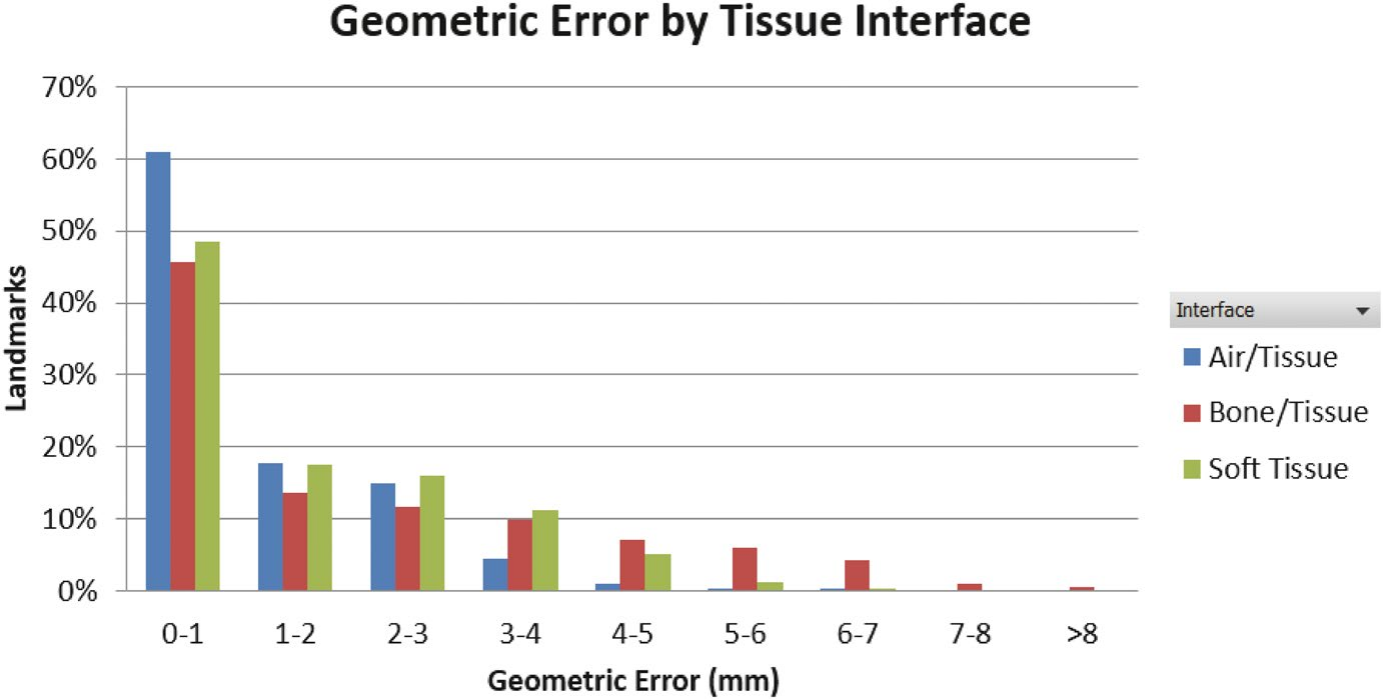
Geometric error histograms from all landmarks of all patients grouped by tissue interface types. All distributions follow the same general shape, with a large peak at 0–1 mm, a smaller peak at 1–2 mm followed by a gradual fall off

**FIGURE 4:**
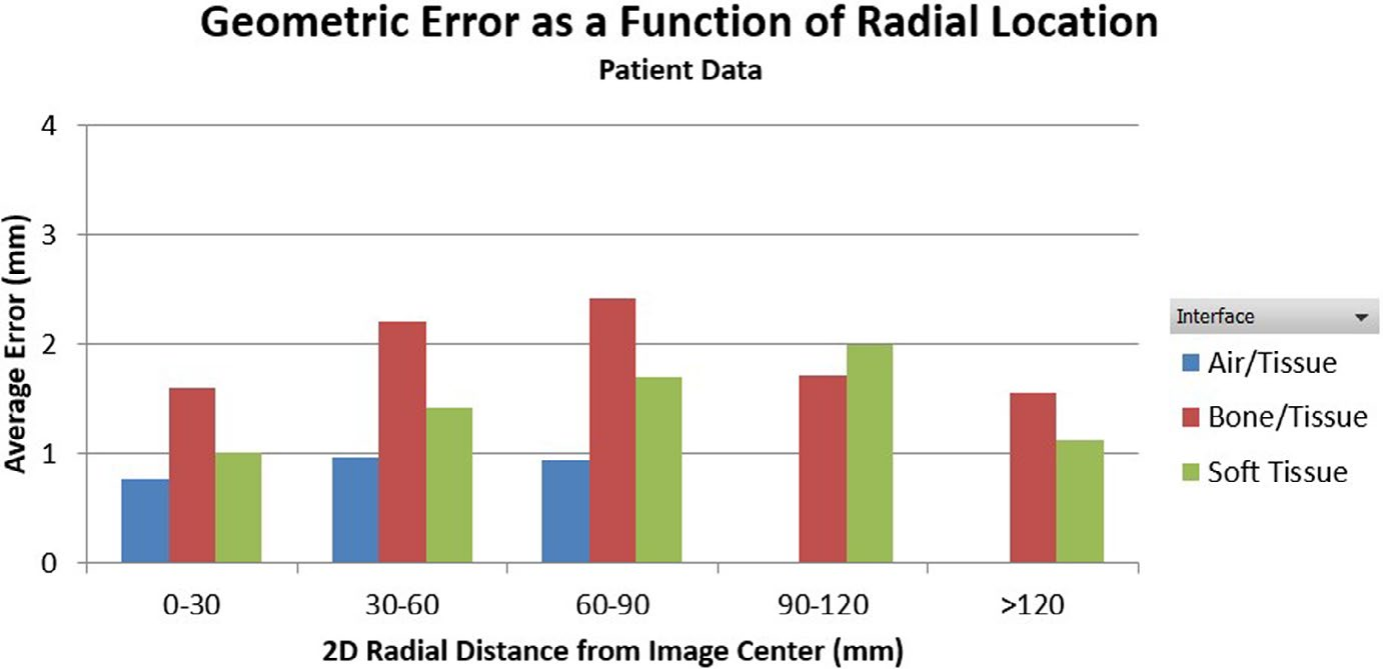
Geometric error plotted as a function of radial location of different tissue interface types for all patients

A total of 970 landmarks (260 for air/tissue, 319 for bone/tissue, and 391 for soft tissues) was identified for HN patients with an average spatial error of 1.46 ± 1.78 mm. Figure 3 shows the histogram of the geometric errors of all landmarks grouped by tissue interface. The histogram peaked at <1 mm error. 66% of the discrepancies were less than 2 mm and 51% less than 1mm. Larger errors were observed at bone/tissue interfaces with an average of 2.01 ± 2.20 mm, compared to 0.88 ± 1.24 mm and 1.41 ± 1.56 mm for the air/tissue and soft tissue, respectively. Statistically significant differences were found between these tissue types with all *p*‐values less than 0.0001. Substantial geometric discrepancies in the range of 7–8 mm were observed at bone/tissue interfaces, although they only constituted a small fraction (0.5%) of all landmarks. The maximum distortion of 8.48 mm was found in the maxillary sinus, on a bone‐tissue interface, and located at 59 mm away from the image center. Within the 5 cm radial FOV, the average discrepancy was 1.13 mm and increased to 1.86 mm from 5 cm to 10 cm FOV. The geometric errors as a function of radial distance to image center were plotted for different tissue interfaces for all patients in Figure 4 and demonstrated a general trend of increasing geometric error with the in‐plane radial distance from the image center.

## 4 DISCUSSIONS

In this study, we performed a comprehensive assessment of the overall spatial distortion of a 0.35 T MR‐guided radiotherapy system by anatomic landmark‐based analysis of MR images compared with corresponding CT as ground truth. The average spatial discrepancies were found to be 1.15 ± 1.14 mm and 1.46 ± 1.78 mm for the anthropomorphic phantom and HN patient data, respectively. Relatively larger errors were found at bone/tissue interfaces compared with the air/tissue and soft tissue interfaces for the patient cohort. The averaged value is slightly higher for the bone‐tissue interface in our study, probably due to the complicated interplay effect of the two major sources of distortion, distance to isocenter and tissue types. We reviewed the distribution of our landmarks and found that most of the bony‐tissue landmarks are located further from the isocenter than the air‐tissue landmarks. In addition, the subject‐induced distortion depends on magnet strength, gradient strength, readout direction, and image sequences. The system‐related distortion also depends on the scanner and vendor‐provided correction algorithm. All these factors make it difficult to directly compare the reported overall distortions between different systems and studies. System‐dependent spatial distortion of the same MRgRT system was evaluated using a 3D grid phantom.^17^ For the same clinical bSSFP sequence, 99.9% of the landmark pointers had a distortion of less than 1mm within 10cm distance to image isocenter and 94.8% were less than 1mm within 17.5cm distance to image isocenter. The average 2D distortions were 0.34, 0.35, and 0.45 mm within 5, 10, and 17.5 cm of image isocenter with maximum distortions of 0.76, 1.15, and 1.88 mm, respectively. Compared with the system‐related distortions, our measurements of the overall spatial distortions were relatively higher, with average 2D distortions of 1.13, 1.86, and 1.53 mm for landmarks within 5 cm, between 5 cm and 10 cm, or greater than 10 cm from image isocenter, demonstrating additional contributions from subject‐dependent distortions induced by magnetic susceptibility and chemical shift effects. The reason that the spatial distortion was slightly lower at >10 cm from the image center could be attributed to the limited number of landmarks identified in this region because of the relatively small size of head and neck anatomy. Directional dependence was also observed with average errors of 1.16 ± 1.63 mm, 0.63 ± 0.92 mm, and 0.04 ± 0.28 mm in the lateral, anterior‐posterior, and superior‐inferior directions, respectively. The directional dependence may be attributed to the frequency encoding direction for the MR imaging sequence used in this study and the uneven distribution of various tissue types across the image.

Our measurements were also in line with published reports assessing patient‐related distortions of other MR systems. Using a similar landmark‐based method, Mohamed et al. evaluated the combined geometric distortion on HN patients scanned on a 3T MR scanner with T2‐FSE sequence.^22^ The median distortion for all the landmarks was 1.06 mm (IQR0.6‐1.98) which is similar to our measurements. It was also observed that the magnitude of distortion was higher in the peripheral region compared to centrally localized landmarks. The maximum distortions of 7–8 mm observed at the peripheral region of Mohamed et al. are also in line with what we have identified. However, tissue interface dependency was not investigated in that study. An in vivo B0 field inhomogeneity mapping method was developed by Wang H et. al. to quantify subject‐induced distortions for 3 T T1 brain images.^19^ Majority of the displacements (97.4%) were found to be <1 mm with only 0.1% >2 mm in the brain region. A maximum distortion of 4mm was identified with most large distortions observed at air–tissue interfaces in the sinuses. Adjeiwaah et al. simulated patient‐induced distortions of HN patients by the bulky assignment of magnetic susceptibility values to segmented tissues on CT images.^25^ The mean patient‐induced distortion was estimated to be 0.76 mm (maximum 2.17 mm) within a radius of 20 cm from the isocenter at 3 T. 15.4% of the voxels were found to have distortions >2 mm and the majority were located close to dental fillings and air–tissue interfaces. The authors also demonstrated that the patient‐induced distortion could be significantly reduced if a higher frequency‐encoding bandwidth was used. It is worth re‐iterating that the subject‐induced distortion depends on magnet strength, gradient strength, and imaging sequence as well as local tissue susceptibility differences, which makes it difficult to directly compare the reported distortions among different systems. The impact of these parameters on susceptibility‐induced distortion was well characterized in a simulation study for various anatomical sites.^21^ The range of the distortion in the brain region was estimated to be 1mm at the air–tissue interface for a 0.5 T magnet compared with 3 mm for a 3 T.

The average spatial discrepancy of 1.46 ± 1.78 mm found in this study indicates that the MR images of the 0.35 T MRgRT system meet the spatial requirement of 2 mm for non‐stereotactic treatment as recommended by AAPM Task Group 142.^5^ In order to meet the 1 mm recommendation for SBRT, the treatment region of interest should be placed as close as possible to the image isocenter where the spatial discrepancy was found to be around 1.1 mm within the 5 cm radial FOV. In our clinic, targets are generally kept within 5 cm of isocenter, although they may occasionally be set greater than 5 cm distance due to the patient positioning limitations. For treatment site close to the image periphery or at different tissue interfaces, large PTV margin may need to be considered. Overall, the observed average level of distortion is small with respect to the 5 mm PTV margins typically used for MRI‐guided SBRT in our clinic; however, further reduction of the margin for the target outside the 5 cm FOV or at tissue interfaces should be explicitly and carefully evaluated. In addition, spatial distortion could also impact dose calculation accuracy. Considering an average distortion of 1.8 mm of soft tissue at an image periphery and approximate attenuation of 1.5% cm^−1^ of a 6 MV beam in water, the corresponding dose error is about 0.3%. A similar dose error of 0.4% of D50 to PTV was observed in a dosimetric study evaluating the impact of MRI distortion of a 3 T scanner on the head and neck treatment planning.^25^

There are some limitations to this study. First, the spatial uncertainties identified in this study may include image registration errors. Even though local image registration was carefully reviewed and adjusted, it is not possible to achieve a perfect image registration, and residual registration errors may lead to the overestimation of the spatial distortion. The anthropomorphic phantom‐based measurement was performed to assess the spatial uncertainties under better control of image registration, which also indicated a slightly smaller average distortion compared to the patient data. Nevertheless, the combined spatial uncertainties observed in patient images are relatively small considering the residual system‐related distortions. Second, only a limited number of HN patients were included in this study. However, a large number of anatomic landmarks were obtained for each patient and a consistent trend was observed among these patients. Third, as with all landmark‐based studies, selection bias and user error are unavoidable. Although the landmarks were selected and reviewed by different oncologists in this study, no inter‐observer variation was quantified which is another limitation of this study and will be evaluated in a future study. It is also worth noting that distortion relies on the location of the region of interest. Geometric distortions for other anatomic sites, such as breast or liver, may still need to be assessed in separate studies.

## 5 CONCLUSION

In this study, the total spatial uncertainties including patient‐dependent distortion of a 0.35 T MRgRT system were evaluated by comparing MR images to CT ground truth images for an anthropomorphic phantom and clinical HN patients. It was found that spatial uncertainty remains in the MR images after systematic distortion corrections are applied. Even though the observed average errors were relatively small and comparable to the recommended spatial accuracy for stereotactic treatment,^5^ large distortions observed at the bone/tissue interface emphasize the need for continued development of quantitative methods for assessing patient‐specific spatial distortions as an important consideration in moving toward MR‐guided radiotherapy practice.

## CONFLICT OF INTERESTS

Y Ying, J Lamb, D Low, A Kishan, and M Cao reported receiving personal fees from ViewRay Inc.

## AUTHOR CONTRIBUTIONS

JN, MC: Study design, data collection and analysis, manuscript writing; KC, YY, DD, KS, RC, AK, JM DL: data collection, manuscript writing.

## Data Availability

Research data are not shared per institution policy.
